# Treatment of Neurocardiogenic Syncope: From Conservative to Cutting-edge

**DOI:** 10.19102/icrm.2018.090702

**Published:** 2018-07-15

**Authors:** Amulya Gampa, Gaurav a. Upadhyay

**Affiliations:** ^1^Department of Internal Medicine, the University of Chicago Medicine, Chicago, IL, USA; ^2^Center for Arrhythmia Care, Section of Cardiology, the University of Chicago Medicine, Chicago, IL, USA

**Keywords:** Autonomic modulation, catheter ablation, ganglion plexus, neurocardiogenic syncope, pacemaker

## Abstract

Neurocardiogenic syncope is the most frequent cause of syncope in the general population. Many years have been spent on determining an effective treatment for this condition. Conventional treatment usually follows a tiered approach for neurocardiogenic syncope, as follows: first, lifestyle modification, including increased fluid intake and the introduction of physical counterpressure maneuvers, is tried; then the use of targeted pharmacologic therapy, particularly agents that support blood pressure or that drive blood pressure is attempted; and, finally, pacemaker implantation in patients with a predominant cardioinhibitory component to their syncopal episodes is performed. More recently, autonomic modulation with cardiac ganglion ablation has emerged as a promising treatment modality for patients refractory to traditional approaches. In this review, we sought to summarize the existing therapies for neurocardiogenic syncope and explore the latest research on new modalities of treatment.

## Introduction

Neurocardiogenic syncope (NCS), also known as vasovagal syncope, is one of the most frequent causes of syncope in the general population. NCS is the most common type of reflex syncope, which also includes carotid sinus and situational syncope. The overall incidence of reflex syncope is 40 per 1,000 patient-years in the general population, although only a subset of these (between 9.3 and 9.5 per 1,000 patient-years) seek medical care for their episodes.^1^ True estimates of overall incidence are likely greater, as isolated syncopal episodes are often under-reported. NCS is typically provoked by stereotypic triggers; these include prolonged standing or sitting upright, intense emotion, pain, and other strong vagal stimulants. Importantly, however, there is also an element of predisposition that makes the onset of NCS more likely for some patients when they are exposed to common triggers. Presentation with NCS usually begins with a prodrome, which may include sensations of dizziness, warmth, diaphoresis, nausea, or palpitations prior to the event. Importantly, some patients—particularly the elderly—may not demonstrate a classical prodrome or may have an atypical presentation.

## Pathophysiology and diagnosis

The mechanism of NCS is not completely understood. Traditionally, the sequence of events leading to syncope is thought to be triggered by venous pooling. This leads to decreased activation of baroreceptors in the aortic arch and carotid sinus, causing increased sympathetic tone. This results in increased ventricular contractility, which in turn activates receptors attuned to wall tension in the left ventricle and thus paradoxically increases vagal output. Increased vagal tone leads to profound vasodilation, which causes syncope. This chain of events is known as the Bezold–Jarisch reflex. It was previously accepted that this reflex caused syncope primarily through a vasodepressor response, but more recent investigation has shown that NCS may be the result of a cardioinhibitory mechanism as well, mediated primarily through bradycardia and an associated reduction in cardiac output. In presyncopal patients, decreased cardiac output has been shown to lead to hypotension. In some individuals, this response can be elicited with nitroglycerin.^2^ Cardiac pacing studies in patients with recurrent syncope have further confirmed the role of bradycardia and cardiac output in NCS.^3^ In addition, the role of sympathetic tone, or lack thereof, in causing vasodilation is unclear. The mechanism of vasodilation and the role it plays in NCS may be more complex than what was initially postulated and requires further investigation.

The diagnosis of NCS is often made through history and physical alone. Further testing may be required when the etiology of syncope is less clear, especially in older patients. Head-up tilt-table testing (HUT) has become a valuable tool for the diagnosis of NCS. HUT began to be widely used for this purpose in the 1980s, following documentation in a report by Kenny et al.^4^ Since then, various protocols for HUT have been devised to aid in the diagnosis of unexplained syncope. Since passive tilt testing alone did not always reliably induce syncope, pharmacologic augmentation was eventually added to most protocols, predominantly using either nitroglycerin or isoproterenol. A meta-analysis of 55 studies comparing the use of HUT in patients with NCS versus in asymptomatic controls showed a diagnostic odds ratio of 12.15 for NCS, with an overall sensitivity of 59% and a specificity of 91%. The administration of nitroglycerin resulted in the highest increase in sensitivity (to 66%), with specificity maintained at 98%.^5^

The Vasovagal Syncope International Study (VASIS) introduced a commonly used classification system for types of vasovagal response after HUT. The modified VASIS classification system is routinely used in clinical practice and identifies three major types of responses: type 1 (mixed), type 2 (cardioinhibition), and type 3 (vasodepressor) **(Table 1)**.^2^ Categorizing patients based on the modified VASIS system has been helpful in identifying treatment approaches used in large trials.

Importantly, however, the value of HUT as a diagnostic test has been brought into question, as it is more likely to be positive in patients with an established diagnosis of NCS than in those with only a possible diagnosis, and the result of tilt testing does not appear to correlate with long-term recurrence of syncope.^6^ In addition, though certain favored HUT protocols exist, there is still significant variation in protocols with regard to timing, tilt angle, and pharmacologic intervention, and there is no consensus as to which protocol demonstrates the highest sensitivity and specificity for diagnosis. More recently, implantable loop recorders (ILRs) have been suggested to aid in the diagnosis and treatment of patients with unexplained syncope, but more study is still required in this area to reach a definitive explanation.^7^

## Need for treatment

The majority of patients with NCS experience only rare, sporadic episodes of such in their lifetime, for which no specific treatment is required. A subgroup of patients, however, will experience recurrent episodes, which can be emotionally distressing or even disruptive to daily life. Although estimates do not exist for the prevalence of recurrent syncope in the general population, it has been shown that individuals are at higher risk of recurrent syncope if they experienced it in the past year.^8^ In patients who do not experience a prodrome, especially in older adults, recurrent syncope can cause falls and physical trauma that can lead to hospitalization and worsened overall morbidity. In light of this, several options for the treatment and prevention of NCS have been evaluated for patients with recurrent syncope. The least-invasive options include lifestyle changes and counterpressure maneuvers, as well as a wide range of pharmacologic therapies. More invasive therapies have been used in patients with syncope due to a predominant cardioinhibitory response. These approaches include pacemaker implantation and, more recently, cardiac ganglion ablation, a new treatment modality that does not require permanent device implantation. This review will provide an overview of the available and developing treatment strategies for NCS.

### Conservative therapy

The initial treatments recommended for patients with recurrent NCS are conservative measures—that is, nonpharmacological and noninvasive therapies that can be easily implemented into patients’ everyday lives. These measures include increasing fluid and salt intake, employing physical countermaneuvers, exercise training, and tilt training. Some of the data supporting these approaches are discussed briefly below.

Increased fluid and salt intake is a frequently suggested first-line intervention for NCS. Volume expansion has been found to be particularly efficacious in patients suffering from orthostatic hypotension but, in patients with NCS, results have been less consistent. Bellard et al. studied a regimented fluid intake protocol in 86 patients suffering from NCS.^9^ The treatment group was instructed to drink 1.5 L of water with 1,500 mg of sodium chloride daily, while the control group did not receive such instructions. After 10 days, no difference was found in the number of positive HUT results in each group. In addition, the investigators found no association between plasma volume and HUT results in either group, suggesting that an increase in the plasma volume may not necessarily prevent NCS.

Physical counterpressure maneuvers (PCMs) are movements such as leg-crossing and hand-gripping motions that are thought to help with preventing the loss of consciousness in patients who can feel preceding presyncopal symptoms. These maneuvers increase systemic vascular resistance and blood pressure, thereby counteracting the vasodepressor effect that leads to syncope. A 2002 study involving 21 patients with recurrent NCS and positive HUT results investigated whether crossing the legs and tensing the muscles during tilt testing would prevent syncope when the prodrome first appeared.^10^ Among 21 patients who performed the maneuvers, symptoms completely resolved in five, while, in the remaining patients, syncope or termination of HUT was postponed by a mean of 2.5 minutes. Another longer-term study examined PCMs in patients with classic NCS by history or possible NCS by history but with positive HUT results.^11^ Patients were randomized to conventional education versus education and training in PCMs, including leg-crossing, hand-gripping, and arm-tensing movements. During an average follow-up period of 14 months, recurrent syncope was found to be significantly reduced with the use of PCMs (ie, the rate of recurrent syncope was 31.6% in the intervention group versus 50.9% in the control group; p = 0.0005). The major limitation to the use of PCMs, however, is that they can only be implemented by patients with a recognizable prodrome.

While HUT has been widely identified as a diagnostic tool for NCS, Di Girolamo et al. also observed that HUT could be used as a therapeutic modality as well.^12^ Various “tilt-training” protocols have since been tested. Foglia-Manzillo et al. evaluated one such tilt-training regimen in patients with recurrent syncope and positive HUT findings.^13^ They were randomized to home tilt training (30 minutes of leaning against a vertical wall) and no tilt training. On repeat HUT three weeks later, no difference was found between the groups in terms of presentation of tilt-provoked syncope. Notably, though, the tilt-training group had a very poor compliance rate. Duygu et al. used a similar protocol in 82 patients, this time measuring spontaneous syncope recurrence rather than tilt-induced syncope.^14^ No significant difference was found between the tilt-training and control groups with regard to the recurrence of spontaneous syncope, time to recurrence, or frequency of syncope after 12 months of follow-up, though the study may have been under-powered to detect a difference.

In an effort to determine if any of these methods lead to a physiologic change that prevents syncope, Gardenghi et al. studied changes in the vagal and sympathetic baroreflexes using microneurography in patients undergoing exercise training (including stretching, cycling, and focal strengthening exercises); tilt training; pharmacological therapy; or no treatment.^15^ They found that only exercise training caused a significant increase in arterial baroreflex sensitivity, indicating the exercise training may be the best way to prevent syncope.

Regardless of the methods chosen for prevention, however, the provision of education is vital in patients experiencing NCS. Counseling regarding the overall benign natural history of classic NCS and the methods of prevention can improve patient quality of life. In an observational study, 316 patients with NCS received education about risks and prognosis, reassurance, and instructions regarding preventative maneuvers (including drinking > 2 L of fluid, avoiding triggers, performing exercise, assuming the supine position when necessary, and countermaneuvers). A significant decrease in syncope burden was observed, in addition to a significant decrease in syncope-related injury.^16^ As evidenced by the above, education can be a simple tool that can make an immense difference in patients’ lives.

### Pharmacologic therapy

When conservative treatment fails or is insufficient in reducing syncope burden, the most reasonable next step for many patients is pharmacologic therapy. As compared with invasive methods of managing syncope, medical therapy is easily implemented, with manageable overall side effect profiles, and modifiable, making it favorable among most practitioners. With that noted, most of the evidence for several classes of medications is weak at best—furthermore, multiple visits to initiate, titrate, or adjust therapy to achieve symptom relief are also often required.

***Midodrine***. Midodrine, a drug whose active metabolite is an alpha-1 adrenergic agonist, may help to prevent NCS through its vasoconstrictive effect. This vasoconstriction can counteract the vasodilation seen in NCS. A few small, randomized controlled trials have shown evidence of symptomatic improvement with midodrine therapy; its use has been indicated to decrease positive HUT results as compared with a placebo^17^ and, perhaps more importantly, Ward et al. showed an improvement in quality of life and an increased number of syncope-free days in patients treated with midodrine versus a placebo.^18^ Similarly, patients on midodrine had improved quality of life and fewer syncopal symptoms in comparison with those treated with fluid and salt tablets.^19^ Overall, the existing evidence suggests a benefit with midodrine use. The Prevention of Syncope Trial (POST) IV trial, which is still ongoing, aims to compare the use of midodrine against a placebo in approximately 140 patients.^20^ This larger, randomized placebo-controlled trial will hopefully provide clearer evidence for the use of midodrine.

The evidence becomes unclear when trials studying etilefrine (not currently available in the United States) are considered. A European study of etilefrine versus a placebo failed to show efficacy of the former in preventing NCS,^21^ but a systematic review studying alpha-adrenergic agonists as a whole showed a decrease in time to first syncope recurrence when compared with standard treatment.^22^ Of note, only some of the studies included showed a significant effect of alpha-adrenergic agents as compared with placebos.

***β-Blockers***. β-Blockers were one of the first medications to be tested as medical therapy for NCS. Given that β-adrenoreceptors partially mediate ventricular baroreflex, which subsequently leads to vasodilation and venous pooling in patients with syncope, investigators postulated that β-blockers would be useful in attenuating this response. After several small studies showed mixed results,^23–25^ POST sought to prove a therapeutic effect to β-blockade.^26^ The randomized, double-blind placebo-controlled trial tested metoprolol in patients aged either < or ≥ 42 years. Neither group showed a significant decrease in the likelihood of syncope when treated with metoprolol.^26^ When these data were combined with those of a different cohort from an observational study, an age-dependent benefit was noted; specifically, syncopal episodes did appear to be reduced in patients aged ≥ 42 years, whereas they were possibly exacerbated in patients who were younger.^27^ A separate, smaller study aiming to elucidate the physiologic effects of β-blockade during syncope demonstrated that propranolol failed to prevent vasodilation, adrenaline release, or syncope during HUT.^28^ A systematic review of 12 trials showed that β-blockers significantly reduced syncope when compared with standard treatment, but did not do so when compared with a placebo.^22^ The lack of effect seen with the use of β-blockers in the comparison with the placebo may be because β-blockers have a significant effect only on older patients and less so on younger patients. Based on the current data, it would be reasonable to treat patients aged 42 years or older with β-blockers, with the caveat that the benefit of such has not yet been proven in this population. The POST IV study seeks to confirm this benefit in older patients.^29^

***Selective serotonin reuptake inhibitors***. In comparison with alpha-adrenergic agonists and β-blockers, the evidence for the use of selective serotonin reuptake inhibitors (SSRIs) is much more limited, although with some favorable findings. Changes in serotonin levels can affect blood pressure and heart rate via the central serotonergic pathway, which can contribute to NCS in some patients. SSRIs may work by stabilizing serotonin levels in the central nervous system. After observational studies reported SSRIs as being effective in preventing syncope, a randomized controlled trial was conducted to specifically test the effectiveness of paroxetine.^30^ Results indeed showed a decrease in both HUT-induced syncope and spontaneous syncope as compared with the placebo. As NCS is frequently linked to episodes of high emotional stress, SSRIs may have a role in preventing syncope through their antidepressant and antianxiety effects. When fluoxetine was compared with propranolol in terms of preventing NCS, fluoxetine was shown to not only decrease the frequency of syncopal episodes but also to improve patient wellbeing and quality of life.^31^

***Fludrocortisone***. Fludrocortisone can be an adjunct to increasing fluid and salt intake in the prevention of syncope. As a mineralocorticoid receptor agonist, it causes reuptake of sodium and water in the kidneys, thus increasing circulating plasma volume. The POST II study studied fludrocortisone against a placebo in patients with NCS (not based on HUT).^32^ The trial was not sufficiently powered to detect a decrease in risk via intent-to-treat analysis, due to the dropout rate and slowed enrollment. However, in post hoc on-treatment analysis, a significant risk reduction was noted in the fludrocortisone group. Few other randomized studies comparing fludrocortisone against a control exist to date. The medication may be useful for treating frequent syncope, but sufficient evidence is not yet available.

### Cardiac pacemaker implantation

In patients with NCS due to a predominant cardioinhibitory component, there are data to suggest that cardiac pacing may be beneficial in preventing syncope. Importantly, pacemaker implantation is an invasive procedure that leaves the patient with a device and leads (with the associated need for future generator changes and the risk of infection). Thus, such should be reserved for patients with severely reduced quality of life due to frequent syncope and who have failed to respond to conservative and medical approaches.

Three open-label trials have suggested that pacemakers could significantly reduce syncope in patients with NCS. The Vasovagal Pacemaker Study (VPS),^33^ which was terminated early, randomized patients to either a pacemaker implantation (DDD with rate-drop response) group or a control group that did not receive pacemakers. Patients were required to have syncope or presyncope with relative bradycardia on HUT to be included in the study. There was an 85.4% relative risk reduction in syncope in the pacemaker group (p < 0.0001). Similarly, the VASIS study^34^ randomized patients with recurrent syncope to receive a pacemaker (DDI with hysteresis) versus no therapy. These patients were more highly selected than those in the previous study; a documented cardioinhibitory response, specifically VASIS class IIa or IIb, was required on a prolonged HUT protocol for patients to be included in the trial. Over a mean of 3.7 years, one out of 19 patients in the pacemaker group had recurrence of syncope, while 14 out of 23 in the control group experienced recurrence (p = 0.0004). Lastly, the Syncope Diagnosis and Treatment trial^35^ compared cardiac pacing to medical therapy. Patients with documented relative bradycardia on HUT were randomized to receive either a pacemaker (DDD with rate-drop response) or therapy with atenolol. Again, the recurrence of syncope was significantly decreased in the pacemaker group (p = 0.004). While the results of these initial trials of pacing for syncope were promising, they all suffered from substantial limitations, including small patient samples, the fact that the incidence of presyncope or changes in the quality of life were not evaluated, and that the results were only applicable to a very select group of patients. Perhaps most importantly, none of the studies were blinded or sham-controlled. It is highly possible that, in the patients who received pacemakers, the surgical procedure itself, rather than cardiac pacing, was enough to create a placebo effect in decreasing the recurrence of syncope.

Two subsequent trials attempted to account for the possible placebo effect of pacemaker implantation. The VPS II trial was the first randomized double-blinded trial to study the effectiveness of pacemakers in NCS.^36^ In the study, patients needed a positive response on HUT (defined as a heart rate × blood pressure of less than 6,000 bpm × mmHg). All patients had a pacemaker implanted, which was set to pacing (DDD with rate-drop response) in the study group or to sensing only (ODO) in the control group. The study found no significant difference in the risk of syncope between the groups. Similarly, the SYNPACE study compared syncope recurrence in patients who received a pacemaker with the pacing function turned on (DDD with rate-drop response) to those who received a pacemaker with the pacing function turned off (OOO).^37^ Like the VPS II study, the results did not indicate a benefit of pacing, though the study was terminated early after only 29 patients were enrolled. Both studies indicated that pacing did not prevent syncopal events, contradicting the results of the initial pacemaker trials. A criticism of VPS II and SYNPACE was noted with respect to patient selection and that the pathophysiology of syncope produced during conventional HUT may not correlate well with that of spontaneous syncope occurring in the same patients.^38^

On the backdrop of these findings, Brignole et al. designed the International Study on Syncope of Uncertain Etiology 2 (ISSUE-2) trial, which selected patients for therapy based on evidence from ILRs.^39^ In a large study of 392 patients with clinically suspected vasovagal syncope, ILRs were placed and patients were followed until the first documented episode of syncope or the end of 24 months—whichever occurred earlier. Only patients who had ILR-documented bradycardia or asystole during spontaneous syncope were selected for pacemaker therapy. In contrast with the findings of VPS and SYNPACE, a significant relative-risk reduction was identified in patients who received pacemakers versus in those who were not subjected to any therapy, demonstrating that the selection of patients with cardioinhibitory syncope using ILRs may better identify patients who will benefit from pacing. An important limitation of ISSUE-2 was that there was no sham-control of patients randomized to no therapy. ISSUE-3 addressed this limitation by again selecting only patients with asystole on ILR and randomizing them to receive a pacemaker with active pacing (DDD with rate-drop response) or sensing only (ODO).^40^ Ultimately, a 57% relative risk reduction was seen in the pacing group versus in the sensing group (p < 0.005), confirming that, in properly selected patients, dual-chamber pacing can reduce the recurrence of syncope, even accounting for the placebo effect. A surprising finding of ISSUE-3 was that patients with positive HUT results, even with documented asystole on ILR, did not appear to benefit from permanent pacemakers, while patients with negative HUT findings did. This was an unexpected result and requires further study.

ILR use may not be the only method available to accurately select patients for therapy. Flammang et al. posit that adenosine triphosphate (ATP) testing is also effective in identifying patients with a dominant cardioinhibitory component to syncope.^41^ They demonstrated that DDD pacing in patients with more than 10 seconds of atrioventricular or sinoatrial pause with ATP administration decreased syncope recurrence as compared with backup atrial pacing. There are currently no other studies, however, that use response to ATP testing as a guide for pacemaker implantation in patients with recurrent NCS.

The data from the above pacemaker trials are summarized in **Table 2**. As a whole, the existing data provide conflicting evidence that cardiac pacing has a role as therapy for recurrent NCS. Two systematic reviews showed a significant effect when pooling all included pacemaker trials, but an analysis of only trials that were double-blinded and which had a sensing-only pacemaker group showed no benefit.^22,42^ Of note, these reviews were both done prior to the ISSUE-3 study. The latest systematic review, which included the recent ISSUE-3 study but excluded the VPS and VPS II studies due to insufficient follow-up time, also showed a benefit in the unblinded studies but not in the blinded trials.^3^ Most reviews suggest that an “expectation effect”—where both the patient and physician are aware of pacemaker implantation—can significantly reduce the occurrence of syncope. Particularly as pacemaker implantation is an invasive, permanent procedure, it should be considered only in patients with the greatest benefit-to-risk ratio (ie, patients older than 40 years of age with recurrent syncope causing injury and decreased quality of life). In addition, several methods of selecting patients, including HUT, ILR, and ATP testing exist and it is still unknown as to which group would benefit the most from pacing. It is clear, however, that only a small, select portion of the overall population would see this benefit. To this effect, the 2017 American College of Cardiology/American Heart Association/Heart Rhythm Society guidelines have designated a class IIb recommendation for dual-chamber pacing in patients older than 40 years of age with recurrent syncope and documented spontaneous pauses correlated with syncope.^43^

## Cardiac ganglion ablation

Most recently, investigators have begun testing a novel approach to treating refractory NCS: radiofrequency catheter ablation. Studies have shown that vagal innervation to the heart is primarily supplied through ganglia located in the atrial wall and specific epicardial fat pads. These cardiac ganglia contain several neuronal cell bodies that innervate the sinoatrial and atrioventricular nodes, thus controlling the parasympathetic innervation to the heart.^44^ In theory, if these ganglia were destroyed via ablation, it would impair the bradycardic response and help to prevent NCS in patients with a primarily cardioinhibitory component. The approach is appealing because it would selectively affect parasympathetic and not sympathetic tone and, although invasive, would not require the use of a permanent device. The cell bodies of parasympathetic nerves are located in the atrial wall and epicardial fat pads, making them easily accessible for ablation, while the cell bodies of sympathetic nerves are located far more proximally in the sympathetic trunk. In light of this, radiofrequency ablation of the atrial wall may destroy sympathetic nerve endings, but these still have the ability to grow back, which is not possible for parasympathetic nerves whose cell bodies have been destroyed.

One of the earliest studies, by Pachon et al., tested this hypothesis using spectral mapping to identify areas with a high concentration of cardiac ganglia. These areas of “fibrillar myocardium” had high fractionated potentials and right-shifted spectra, reflecting areas in which nerve fibers were interweaved with myocardial cells.^45^ These were the sites chosen for ablation. Anatomically-guided ablation was also performed, with targets in the regions of the epicardial fat pads known to contain paracardiac ganglia. Of five patients with severe NCS who underwent the procedure, none experienced a recurrence of syncope during a follow-up period of nine months. In a subsequent study by the same investigators, 43 patients with NCS underwent the same procedure and the results were deemed promising: 40 patients had no syncope recurrence during the mean follow-up period of 45 months, while 38 were completely symptom-free.^46^

Several other case reports and case series have also suggested a beneficial effect of cardiac ganglion ablation, but it is unclear how long these effects last and what method of identifying sites for ablation yields the best results. A case report of a 15-year-old female who underwent ablation of the superior and inferior cardiac ganglia in the posterior interseptal area remained syncope-free for nine months, but then had three recurrences in the following four months, though with a longer prodrome.^47^ A later study followed 10 patients who were treated with radiofrequency ablation of up to four ganglionated plexus sites in the left atrial endocardium. None had recurrence of spontaneous syncope in the follow-up period of 13 months to 55 months; however, follow-up tilt testing at three months was positive for syncope in four patients.^48^ Both of these studies used high-frequency stimulation in areas known to have ganglionated plexi to determine sites of ablation.

Other studies did not use high-frequency stimulation but rather chose sites based on anatomic location as determined in previous studies. Rebechi et al. chose to ablate in a “cloud-like” fashion in three predetermined areas in the right atrium, with the assumption that this method of ablation would likely target the desired cardiac ganglia without the need for spectral mapping or high-frequency stimulation.^49^ The two patients who were studied did not have recurrence of syncope in eight and five months, respectively, but one patient did experience three episodes of presyncope at five months. Debruyne et al. chose to target only the anterior right ganglionated plexus with three ablations, with a goal of achieving a “tailored vagolysis” of the sinus node in a 16-year-old female. She had no further episodes of syncope in the following 22 months.^50^ Aksu et al. used a combination of spectral mapping (as described by Pachon et al.^45^) and high-frequency stimulation to localize three specific paracardiac ganglia for ablation in patients with vasovagal syncope, functional atrioventricular block, or sinus node dysfunction.^51^ In the vasovagal syncope group (eight patients), there was no recurrence of syncope within 12 months.

**Figure 1:**
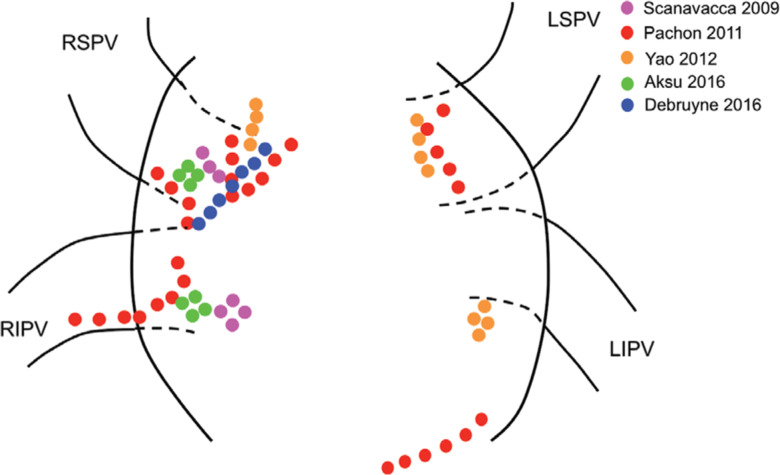
An approximation of left atrial ablation sites in cardiac ganglion ablation studies. The colored dots represent ablation sites. LIPV: left inferior pulmonary vein; LSPV: left superior pulmonary vein; RIPV: right inferior pulmonary vein; RSPV: right superior pulmonary vein.

**Figure 2:**
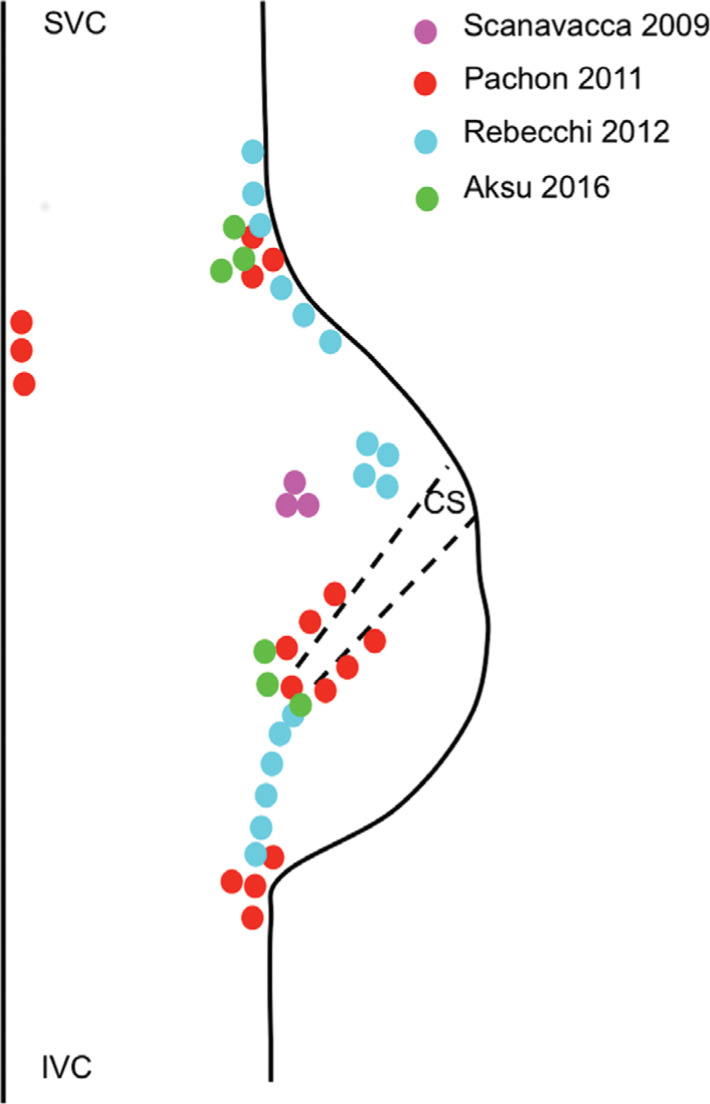
An approximation of right atrial ablation sites in cardiac ganglion ablation studies. The colored dots represent ablation sites. Septal and right atrial lesions were delivered posterior to the atrioventricular node. CS: coronary sinus; IVC: inferior vena cava; SVC: superior vena cava.

The results of the existing studies suggest that the ablation of cardiac ganglia may provide long-lasting effects for patients suffering from refractory syncope, but it is important to keep in mind that these are only preliminary studies for a new procedure. The results of these studies are summarized in **Table 3**. All of the studies are limited by small patient samples and a lack of long-term follow-up data. Furthermore, several questions still need to be answered. Spectral mapping, high-frequency stimulation, the approximation of anatomical sites, or combinations of these three have all been used to identify sites for ablation. It remains to be seen which best prevents syncope. In addition, the location, number, and size of the sites ablated varied among all of the studies **(Figures 1 and 2)**. It appears that more and larger areas of ablation would more effectively prevent syncope, but the adverse effects, especially in the long-term, are still unknown. Randomized controlled trials are still required to identify if a true benefit of cardiac ganglion ablation even exists. As previously shown with the pacemaker trials, a placebo effect could certainly be impacting patients’ experience of symptoms, simply by virtue of them undergoing an invasive procedure. As this burgeoning area of research continues to grow, it is hopeful that many of these questions will be answered in the near future.

## Our approach

**Figure 3:**
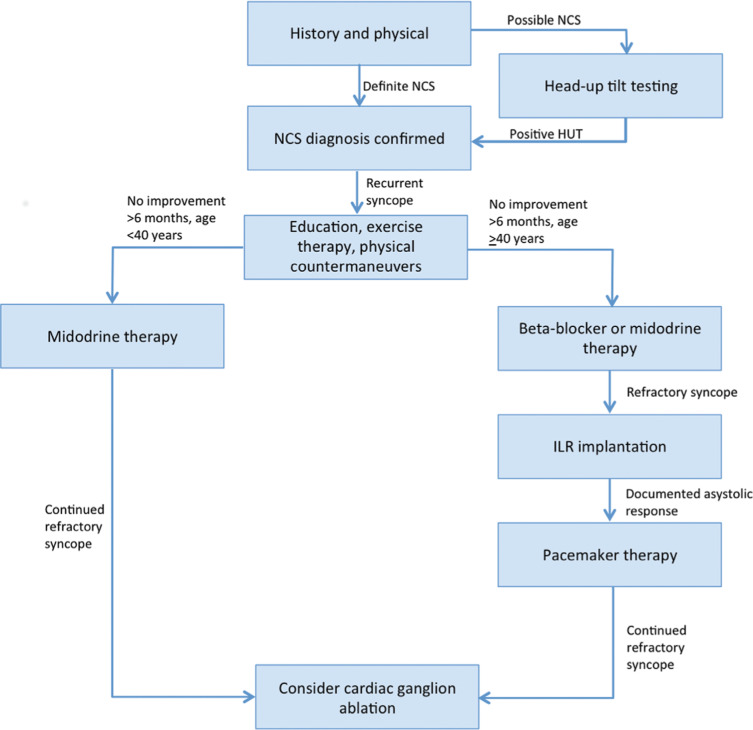
Our recommended approach to patients with NCS.

We suggest the following approach to treating patients with recurrent NCS **(Figure 3)**: (1) the diagnosis of NCS should be verified by a thorough patient history and physical. If the diagnosis is still uncertain, HUT should be performed with and without isoproterenol or nitroglycerin augmentation to induce syncope. Patients with recurrent NCS (we suggest a cutoff criterion of two or more episodes in two years) may be treated if syncope results in injury or if quality of life is affected. (2) All patients should be educated regarding the benign nature of syncope and should be given reassurance. PCMs should be taught to all patients and exercise training should be provided to patients who can tolerate such. (3) For patients who do not experience an improvement in symptoms after at least six months using the conservative measures above, medications should be prescribed. We suggest midodrine in patients aged younger than 40 years of age and a β-blocker (ie, metoprolol) in patients aged older than 40 years of age. Midodrine may also be used in patients aged older than 40 years of age who cannot tolerate or who fail to show benefit with β-blocker therapy. (4) In patients aged older than 40 years of age with refractory syncope on medical treatment, an ILR should be placed. Patients who have syncopal episodes correlating with ILR-documented asystole should undergo dual-chamber pacemaker implantation (DDD with rate-drop response). Lastly, (5) patients who fail all of the above therapies should be considered as candidates for cardiac ganglion ablation.

## Conclusions

The pathophysiology of NCS is yet to be fully elucidated. In most cases, it is a benign condition, but, in those who experience recurrent syncope or who are at a higher risk of physical trauma, treatments can improve the quality of life and prevent harm. Several therapeutic options are available, including conservative measures, midodrine and β-blocker therapy, and more invasive methods such as pacemaker implantation. Cardiac ganglion ablation is also a potential option for treating primarily cardioinhibitory syncope and may be favorable as compared with pacemaker implantation, as it does not mandate permanent device implantation. Ablation for NCS, however, requires further randomized study in larger patient populations to determine overall benefit, appropriate patient selection, and long-term risk and recurrence profiles.

## Figures and Tables

**Table 1:** Modified VASIS Classification of Syncope

Class	Name	Definition	
Type I	Mixed	•	Heart rate falls to no less than 40 bpm, with or without asystole of less than three seconds
		•	Blood pressure falls before heart rate
Type IIa	Cardioinhibition without asystole	•	Heart rate falls to less than 40 bpm for more than 10 seconds, without asystole of more than three seconds
		•	Blood pressure falls before heart rate
Type IIb	Cardioinhibition with asystole	•	Asystole of more than three seconds occurs
		•	Heart rate and blood pressure fall together or heart rate falls before blood pressure
Type III	Vasodepressor	•	Heart rate does not fall more than 10% from peak heart rate
Exception I	Chronotropic incompetence	•	No rise in heart rate during tilt
Exception II	Excessive heart rate increase	•	Excessive increase in heart rate in upright position

bpm: beats per minute.

**Table 2:** Trials of Pacemakers for NCS

Study	Type of Study	Inclusion Criteria	Number of Patients (Intervention/Control)	Intervention/Control	Mean Follow-up	Syncope Recurrence (Intervention/Control)
Connolly et al. 1999^33^	Nonblinded RCT	≥ six episodes of syncope plus HUT with relative bradycardia	54 (27/27)	DDD with rate-drop response/standard treatment	1 year	22%/70% (p < 0.0001)
Sutton et al. 2000^34^	Nonblinded RCT	≥ three episodes of syncope in two years; VASIS IIa or IIb on HUT	42 (19/23)	DDI with hysteresis/no therapy	3.7 years	5%/61% (p = 0.0006)
Ammirati et al. 2001^35^	Nonblinded RCT	≥ three episodes of syncope in two years plus HUT with relative bradycardia	93 (46/47)	DDD with rate-drop response/atenolol	520 days	4.3%/25.5% (p = 0.004)
Connolly et al. 2003^36^	Double-blinded RCT	≥ six total episodes of syncope or ≥ three episodes in two years plus HUT with heart rate × blood pressure < 6,000 bpm × mmHg	94 (42/52)	DDD with rate-drop response/sensing only (ODO)	6 months	33%/42% (p = 0.14)
Raviele et al. 2004^37^	Double-blinded RCT	≥ six episodes of syncope plus HUT with asystolic or mixed response	29 (16/13)	DDD with rate-drop response/no pacing (OOO)	715 days	50%/33% (p = not reported)
Brignole et al. 2006^39^	Observational	≥ three episodes of syncope in two years	392	Implantable loop recorder- based specific therapy (pacemaker, anti-tachycardia therapy)/no therapy	9 months	11%/35% (p = 0.002)
Brignole et al. 2014^40^	Double-blinded RCT	≥ three episodes of syncope in two years; ILR-documented asystolic response	77 (38/39)	DDD with rate-drop response/sensing only (ODO)	2 years	25%/57% (p = 0.039)
Flammang et al. 2012^41^	SIngle-blinded RCT	Isolated or recurrent syncope; > 10-second pause on ATP test	80 (39/41)	DDD/backup pacing (AAI)	16 months	66%/21% (p = not reported)

NCS: neurocardiogenic syncope; RCT: randomized controlled trial; HUT: head-up tilt test; ILR: implantable loop recorder; ATP: adenosine triphosphate.

**Table 3:** Cardiac Ganglion Ablation Studies

Study	Number of Patients	Type of Study	Method of Site Selection	Areas of Ablation	Follow-up Period/Syncope Recurrence
Pachon et al. 2011^46^	43	Prospective cohort	Spectral mapping and anatomic	Areas of fibrillar myocardium (identified by spectral mapping) in the LA and RA; anatomic endocardial ablation of epicardial fat pads in the area between the aorta and SVC, between the right PVs and the RA, and the inferoposterior interatrial septum	22 months/3 patients had syncope recurrence
Scanavacca et al. 2009^47^	1	Case report	HFS	Right superior and inferior ganglia of the posterior interseptal area	12 months/3 recurrences
Yao et al. 2012^48^	10	Case series	HFS	Sequential ablation of four typical ganglionated plexi sites in the LA near the ostia of the PVs: between the LSPV and the LA, inferior to the LIPV, anterior to the RSPV, and inferior to the RIPV	13–55 months/no syncope recurrence
Rebecchi et al. 2012^49^	2	Case series	Anatomic sites in the RA where ganglionated plexi are considered highly probable; ablation performed in “cloud-like” fashion	Superoposterior, posteromedial, and inferoposterior areas of the RA	Case 1: 8 months/no syncope recurrence Case 2: 5 months/no syncope recurrence
Debruyne et al. 2016^50^	1	Case report	Anatomic ablation of anterior right ganglionated plexus	Anterosuperior part of junction between the RSPV and the LA	22 months/no syncope recurrence
Aksu et al. 2016^51^	22	Case series	Ablation of 3 anatomic sites (same as in Pachon et al.^45^), guided by spectral mapping with confirmatory HFS. Started in LA and moved to RA	Area between the aorta and the SVC, between the right PVs and the RA, and at the inferoposterior interatrial septum (same as Pachon et al.^46^)	10.9 months/1 patient with syncope recurrence (0 patients in NCS group)

LA: left atrium; RA: right atrium; SVC: superior vena cava; PVs: pulmonary veins; HFS: high-frequency stimulation; LSPV: left superior pulmonary vein; LIPV: left inferior pulmonary vein; RSPV: right superior pulmonary vein; RIPV: right inferior pulmonary vein.
